# Entanglement Dynamics Induced by a Squeezed Coherent Cavity Coupled Nonlinearly with a Qubit and Filled with a Kerr-Like Medium

**DOI:** 10.3390/e23050496

**Published:** 2021-04-21

**Authors:** Abdel-Baset A. Mohamed, Hichem Eleuch

**Affiliations:** 1 Department of Mathematics, College of Science and Humanities in Al-Aflaj, Prince Sattam Bin Abdulaziz University, Al-Aflaj 11942, Saudi Arabia; 2 Department of Mathematics, Faculty of Science, Assiut University, Assiut 71515, Egypt; 3 Department of Applied Physics and Astronomy, University of Sharjah, Sharjah 27272, United Arab Emirates; heleuch@sharjah.ac.ae; 4 Department of Applied Sciences and Mathematics, College of Arts and Sciences, Abu Dhabi University, Abu Dhabi 59911, United Arab Emirates; 5 Institute for Quantum Science and Engineering, Texas A&M University, College Station, TX 77843, USA

**Keywords:** qubit-cavity detuning, Kerr-like medium, phase-damping, entanglement

## Abstract

An analytical solution for a master equation describing the dynamics of a qubit interacting with a nonlinear Kerr-like cavity through intensity-dependent coupling is established. A superposition of squeezed coherent states is propped as the initial cavity field. The dynamics of the entangled qubit-cavity states are explored by negativity for different deformed function of the intensity-dependent coupling. We have examined the effects of the Kerr-like nonlinearity and the qubit-cavity detuning as well as the phase cavity damping on the generated entanglement. The intensity-dependent coupling increases the sensitivity of the generated entanglement to the phase-damping. The stability and the strength of the entanglement are controlled by the Kerr-like nonlinearity, the qubit-cavity detuning, and the initial cavity non-classicality. These physical parameters enhance the robustness of the qubit-cavity entanglement against the cavity phase-damping. The high initial cavity non-classicality enhances the robustness of the qubit-cavity entanglement against the phase-damping effect.

## 1. Introduction

The study of quantum coherence dynamics induced by open nonlinear qubit-photon systems has recently become a significant area that contributes to the development of potential quantum information applications [1,2,3,4,5,6]. We can cite as examples of potential applications in quantum teleportation protocol [7], cryptography [8], and computation [9,10], generation of entangled states [11]. Entanglement is an essential quantum information resource in quantum communication [12], it has been extensively explored theoretically and experimentally [13,14]. For pure states and closed quantum systems, the entropy is a good measure of entanglement [15]. However, for the mixed states and open systems (specially qubit-photon system that has (2⊗n)-quantum state), the entanglement dynamics can be quantified by the negativity and concurrence.

Interaction of a qubit with a nonclassical light cavity field has fundamental and practical roles in the enhancement or generation of quantum effects [16,17] as quantum coherence and quantum correlations. Therefore, non-classicality in the cavity field needs to be explored further for the quantum information resources. An important resource of the cavity field non-classicality is the squeezed coherent state that was proposed as extension for the coherent state and has essential contributions in quantum optics [17,18,19]. The key feature that distinguishes quantum from classical is the superposition of quantum states. The superposition of coherent state is potentially useful in many quantum information applications [20,21,22,23]. The superposition of coherent state has been experimentally realized in several systems such as superconducting Josephson junctions, [24,25] and linear optical systems [26]. We suggest here a new superposition scheme of squeezed coherent states to prepare a nonclassical light cavity field with a strong non-classicality.

Resources of the nonlinearity effects in qubit-photon interactions (including Kerr-like nonlinearity and intensity-dependent coupling) were realized experimentally in artificial qubit systems as superconducting circuits [27,28], optomechanical systems [29]. The nonlinearity effects have the ability to excite the qubit-photon interactions to improve the generated quantum coherence [30,31]. The qubit-field interactions are generalized to intensity-dependent qubit-field coupling [32]. The effects of the intensity-dependent coupling on the non-classical effects have been studied in a hybrid Cooper pair box qubit interacting with a resonator [33,34]. It is used to enhance the non-local correlations of two coupled qubits [35].

In open systems, the quantum qubit-photon coherence dynamics suffer from the irreversible effects [36,37,38,39] as: dissipation and decoherence. Different types of damping for the qubit and the cavity arise from these dissipation/decoherence effects, such as: amplitude damping, phase-damping, and thermal damping. There are several approaches to describe the effects of the coupling between the system and its surrounding reservoir. For diagonal effective Hamiltonian, qubit-photon coherence dynamics has been studied for a phase and thermal damped cavity [40]. The dynamics of quantum correlations in several qubit-photon systems were investigated without the effects the coupling to the surrounding environment [41,42,43,44]. The method used in this paper can be used to investigate the dynamics of quantum information resources in other qubit-photon structures while taking into account the implications of coupling to the external environment.

In this paper, we analyze the effects of intensity-dependent coupling, Kerr nonlinearity, phase cavity damping and qubit-cavity detuning on the dynamics of the qubit-cavity entanglement. The paper is organized as follow; In the Section 2, the model of dissipative nonlinear qubit-cavity interactions is presented with its analytical solutions. In Section 3, the dynamics of the generated qubit-cavity entanglement are investigated. We end up with a conclusion.

## 2. Physical Model

Here, the proposed physical model is motivated by the realizations of: (1) the nonlinearity in artificial qubit system [45], for examples: Resonator self-Kerr-nonlinearity was realized with the SQUID cosine potential to include nonquadratic corrections [28]. (2) The intensity-dependent coupling was constructed based on a Cooper-pair box with a superconducting loop embedded in a microwave cavity field [32,46]. Therefore, we consider a two-level atom (qubit with the upper state |1〉 and the lower |0〉 states) interacting off-resonantly with a dissipative nonlinear Kerr-like cavity through intensity-dependent coupling. We assume that the environment is at zero temperature and the system decoherence is due to a phase-damping reservoir. This interaction preserves the energy of the system. The Hamiltonian of the system-reservoir is,

(1) $$\begin{array}{ccc} {\hat{H}}_{SR} & = & \omega_{f}{\hat{\psi }}^{†}\hat{\psi }+\omega_{q}\frac{\sigma_{z}}{2}+\chi {\hat{\psi }}^{2†}{\hat{\psi }}^{2}+\sum_{i}\omega_{i}{\hat{\pi }}_{i}^{†}\;{\hat{\pi }}_{i}+\lambda (\hat{\psi }Y\left({\hat{\psi }}^{†}\hat{\psi }\right)|1\rangle \langle 0|\\ \; & \; & \;\;+Y\left({\hat{\psi }}^{†}\hat{\psi }\right){\hat{\psi }}^{†}|0\rangle \langle 1|)+\eta_{i}\left({\hat{\pi }}_{i}^{†}{\hat{\psi }}^{†}\hat{\psi }+{\hat{\psi }}^{†}\hat{\psi }{\hat{\pi }}_{i}\right). \end{array}$$

Here ${\hat{\psi }}^{†}\left(\hat{\psi }\right)$ represents the cavity field operators, and λ is the qubit-cavity coupling. $\omega_{q}$ and $\sigma_{z}=\left|1\rangle \langle 1\right|-\left|0\rangle \langle 0\right|$ are the frequency and the population inversion operator of the qubit. Where ${\hat{\pi }}_{i}$ and $\omega_{i}$ denote the operators and frequencies of *i*-th bath oscillators, and $\eta_{i}$ represents the system-bath coupling strength. χ>0 is the Kerr-like nonlinearity, i.e., the two-photon coupling strength proportional to the third-order nonlinear susceptibility. However, $\hat{Y}\left({\hat{\psi }}^{†}\hat{\psi }\right)$ denotes the function that describes the intensity-dependent qubit-field coupling, if $\hat{Y}\left({\hat{\psi }}^{†}\hat{\psi }\right)=\hat{I}$ and χ=0, the Hamiltonian reduces to that of the standard Jaynes-Cummings model.

The dynamics of the qubit-cavity state $\hat{R}\left(t\right)$ under the phase cavity damping is explored by [47]

(2) $$\begin{array}{ccc} \frac{d\hat{R}\left(t\right)}{dt} & = & -i[\hat{H},\hat{R}]+\gamma \left(\left[{\hat{\psi }}^{†}\hat{\psi }\hat{R}\left(t\right),{\hat{\psi }}^{†}\hat{\psi }\right]+\left[{\hat{\psi }}^{†}\hat{\psi },\hat{R}\left(t\right){\hat{\psi }}^{†}\hat{\psi }\right]\right), \end{array}$$

where the system Hamiltonian is given by: $H=\omega_{f}\left({\hat{\psi }}^{†}\hat{\psi }+\frac{1}{2}\sigma_{z}\right)+\frac{\Delta }{2}\sigma_{z}+\chi {\hat{\psi }}^{2†}{\hat{\psi }}^{2}$$+\lambda (\hat{\psi }Y\left({\hat{\psi }}^{†}\hat{\psi }\right)|1\rangle \langle 0|+h.c.)$. $\Delta =\omega_{q}-\omega_{f}$ represents the qubit-cavity rate. $\gamma =\eta (2\bar{n}+1)$ is the phase-damping with a mean occupation number $\bar{n}$, and $\eta_{i}=\eta$ is the coupling to the reservoir of oscillators [48].

To find analytical solution of Equation (2) in the strong coupling regime, we use the dressed-states method [49]. The Hamiltonian preserves the number of excitations in the system (which is not the same as preservation of energy). This allows one to look for solutions separately in each manifold with a fixed number of excitations, i.e., in the form of superpositions of the eigenstates $|E_{n}^{\pm }\rangle$. In the qubit-cavity basis: {|i,n〉}(i=0,1,n=0,1,2,..., and |n〉 is the number state), the eigenstates $|E_{n}^{\pm }\rangle$ and the eigenvalues $V_{n}^{\pm }$ of the system Hamiltonian *H* are

$$\begin{array}{ccc} |E_{0}\rangle & = & |0,0\rangle ,\;V_{0}=-\frac{\omega_{q}}{2}, \end{array}$$

(3) $$\begin{array}{ccc} |E_{n}^{\pm }\rangle & = & X_{n}^{\pm }|1,n\rangle \pm X_{n}^{\mp }|0,n+1\rangle ,\; \end{array}$$

(4) $$\begin{array}{ccc} V_{n}^{\pm } & = & \omega \left(n+\frac{1}{2}\right)+\frac{1}{2}\left[K_{n}+K_{n+1}\right]\pm \Lambda_{n},\\ \Lambda_{n} & = & \sqrt{{\left[\delta +K_{n}-K_{n+1}\right]}^{2}+\lambda^{2}\left(n+1\right)Y^{2}\left(n\right)}, \end{array}$$

with the Kerr-like nonlinearity function: $K_{n}=n^{2}\chi -n\chi$ and

$$\begin{array}{ccc} X_{n}^{\pm } & = & \frac{1}{\sqrt{2}}\sqrt{1\pm \frac{\delta +K_{n}-K_{n+1}}{\Lambda_{n}}}. \end{array}$$

We apply now the canonical transformation,

(5) $$\begin{array}{c} \dot{A}\left(t\right)=\frac{\partial }{\partial t}\left\{e^{i\hat{H}t}\hat{R}\left(t\right)e^{-i\hat{H}t}\right\}, \end{array}$$

then in the basis: $\{{\hat{S}}_{11}^{m}=|E_{m}^{+}\rangle \langle E_{m}^{+}|,{\hat{S}}_{12}^{m}=|E_{m}^{+}\rangle \langle E_{m}^{-}|,{\hat{S}}_{21}^{m}=|E_{m}^{-}\rangle \langle E_{m}^{+}|,{\hat{S}}_{22}^{m}=|E_{m}^{-}\rangle \langle E_{m}^{-}|\}$, the Equation (2) takes the form

(6) $$\begin{array}{ccc} \dot{A}\left(t\right) & = & 2\gamma \sum_{m,n=0}^{\infty }\left(T_{m}{\hat{S}}_{11}^{m}+G_{j}{\hat{S}}_{11}^{m}\right)A\left(T_{n}{\hat{S}}_{11}^{n}+G_{n}{\hat{S}}_{11}^{n}\right)\\ & & +D_{m}D_{n}\left({\hat{S}}_{12}^{m}A{\hat{S}}_{21}^{n}e^{2i\beta_{mn}t}+{\hat{S}}_{21}^{m}A{\hat{S}}_{12}^{n}e^{-2i\beta_{mn}t}\right)\\ & & -\gamma \sum_{m=0}^{\infty }\left(T_{m}^{2}+D_{m}^{2}\right)\left({\hat{S}}_{11}^{m}A+A{\hat{S}}_{11}^{m}\right)+\left(G_{m}^{2}+D_{m}^{2}\right)\left({\hat{S}}_{22}^{m}A+A{\hat{S}}_{22}^{m}\right), \end{array}$$

where $T_{m}=m+{\left(X_{m}^{-}\right)}^{2}$, $\beta_{mn}=\Lambda_{m}-\Lambda_{n}$, $G_{m}=m+{\left(X_{m}^{+}\right)}^{2}$, and $D_{m}=X_{m}^{+}X_{m}^{-}$.

To find analytical solutions, we consider that the initial state of the system is given by

(7) $$\begin{array}{ccc} \hat{R}\left(0\right) & = & A\left(0\right)=\sum_{i,j=0}P_{i}P_{j}\left|1,i\rangle \langle 1,j\right|=\sum_{i,j=0}P_{i}P_{j}\;\;[X_{i}^{+}X_{j}^{+}|E_{i}^{+}\rangle \langle E_{j}^{+}|\\ \; & \; & +X_{i}^{+}X_{j}^{-}|E_{i}^{+}\rangle \langle E_{j}^{-}|+h.c.], \end{array}$$

The upper state |1〉 is considered to be the initial state for the qubit. The initial cavity field is a superposition of coherent squeezed coherent states (SCs) $|\zeta_{S}\rangle$, which is the most general Gaussian pure state. It is defined as

(8) $$\begin{array}{ccc} |\zeta_{S}\rangle & = & \sqrt{N}[|\zeta ,r\rangle +s|-\zeta ,r\rangle ]=\sum_{n=0}^{\infty }P_{n}|n\rangle , \end{array}$$

where the coherent squeezed coherent state [18] is given by

(9) $$\begin{array}{ccc} |\zeta ,r\rangle & = & \sum_{n=0}^{\infty }\frac{1}{\sqrt{n!\alpha }}{\left(\frac{\beta }{2\alpha }\right)}^{\frac{n}{2}}\;e^{-\frac{1}{2}{\left|\zeta \right|}^{2}+\frac{\beta }{2\alpha }\zeta^{2}}H_{n}\left(\frac{\zeta }{\sqrt{2\beta \alpha }}\right)|n\rangle . \end{array}$$

We have used here $\alpha =\cosh r,\;\;\beta =e^{i\theta }\;\sinh r$. $H_{n}\left(x\right)$ is the Hermite polynomial of the order *n*. where ${\left|\zeta \right|}^{2}$ represents the initial mean photon number operator, and *s* is the SCs parameter. The SCs can be reduced to squeezed coherent state for s=0. Even squeezed coherent state is realized for s=1. The normalization factor *N* has the following expression,

(10) $$N=\frac{1}{\left[1+s^{2}+2s\langle -\zeta ,r|\zeta ,r\rangle \right]}.$$

By using the initial density matrix A(0) of Equation (12) and applying the strong coupling approximation (γ≪λ) (i.e., we neglect the oscillatory terms from Equation (5)), then the elements $H_{mn}^{\epsilon \kappa }=\langle E_{m}^{\epsilon }\left|A\left(t\right)\right|E_{n}^{\kappa }\rangle$ (for all ϵ=±, κ=±, and for m≠n) of the density matrix A(t) are given by

(11) $$\begin{array}{ccc} H_{mn}^{++} & = & P_{m}P_{n}X_{m}^{+}X_{n}^{+}e^{-\gamma t[{\left[m-n+X_{m}^{+2}-X_{n}^{+2}\right]}^{2}+{\left(X_{m}^{+}X_{n}^{+}\right)}^{2}+{\left(X_{n}^{+}X_{m}^{+}\right)}^{2}]},\\ H_{mn}^{--} & = & P_{m}P_{n}X_{m}^{-}X_{n}^{-}e^{-\gamma t[{\left[m-n+X_{m}^{-2}-X_{n}^{-2}\right]}^{2}+{\left(X_{m}^{+}X_{n}^{+}\right)}^{2}+{\left(X_{n}^{+}X_{m}^{+}\right)}^{2}]},\\ H_{mn}^{+-} & = & P_{m}P_{n}X_{m}^{+}X_{n}^{-}e^{-\gamma t[{\left[m-n+X_{m}^{+2}-X_{n}^{-2}\right]}^{2}+{\left(X_{m}^{+}X_{n}^{+}\right)}^{2}+{\left(X_{n}^{+}X_{m}^{+}\right)}^{2}]},\\ H_{mn}^{-+} & = & P_{m}P_{n}X_{m}^{-}X_{n}^{+}e^{-\gamma t[{\left[m-n+X_{m}^{-2}-X_{n}^{+2}\right]}^{2}+{\left(X_{m}^{+}X_{n}^{+}\right)}^{2}+{\left(X_{n}^{+}X_{m}^{+}\right)}^{2}]}. \end{array}$$

For m=n, the elements $H_{n}^{\epsilon \kappa }=\langle E_{n}^{\epsilon }\left|A\left(t\right)\right|E_{n}^{\kappa }\rangle$ (for only ϵ=κ=±) verify

(12) $$\begin{array}{ccc} {\dot{H}}_{n}^{\pm \pm } & = & 2\gamma {\left(X_{n}^{+}X_{n}^{-}\right)}^{2}H_{n}^{\mp \mp }-2\gamma {\left(X_{n}^{+}X_{n}^{-}\right)}^{2}H_{n}^{\pm \pm }. \end{array}$$

Now, we use the expressions of the elements $H_{mn}^{\epsilon \kappa }$ of the density matrix A(t) and the inverse canonical transformation of Equation (5): $\hat{R}\left(t\right)=e^{-i\hat{H}t}A\left(t\right)e^{i\hat{H}t}$, to determine the solution of the master equation in the basis: {|i,n〉}(i=0,1) which is:

(13) $$\begin{array}{ccc} \hat{R}\left(t\right) & = & \sum_{m,n=0}\left[X_{mn}^{++}{\tilde{H}}_{mn}^{++}+X_{mn}^{++}{\tilde{H}}_{mn}^{+-}+X_{mn}^{++}{\tilde{H}}_{mn}^{-+}+X_{mn}^{-+}{\tilde{H}}_{mn}^{--}\right]\left|1,n\rangle \langle 1,n\right|\\ \; & + & \left[X_{mn}^{+-}{\tilde{H}}_{mn}^{++}+X_{mn}^{--}{\tilde{H}}_{mn}^{+-}-X_{mn}^{++}{\tilde{H}}_{mn}^{++}-X_{mn}^{-+}{\tilde{H}}_{mn}^{--}\right]\left|1,n\rangle \langle 0,n+1\right|\\ \; & + & \left[X_{mn}^{-+}{\tilde{H}}_{mn}^{++}-X_{mn}^{++}{\tilde{H}}_{mn}^{+-}+X_{mn}^{--}{\tilde{H}}_{mn}^{-+}-X_{mn}^{+-}{\tilde{H}}_{mn}^{--}\right]|0,n+1\rangle \langle 1,n|\;\\ \; & + & \left[X_{mn}^{--}{\tilde{H}}_{mn}^{++}-X_{mn}^{+-}{\tilde{H}}_{mn}^{+-}-X_{mn}^{-+}{\tilde{H}}_{mn}^{-+}+X_{mn}^{-+}{\tilde{H}}_{mn}^{--}\right]|0,n+1\rangle \langle 0,n+1|. \end{array}$$

where $X_{mn}^{\epsilon ,\kappa }=X_{m}^{\epsilon }X_{n}^{\kappa }$ and ${\tilde{H}}_{mn}^{\epsilon ,\kappa }=H_{mn}^{\epsilon \kappa }e^{-i(E_{m}^{\epsilon }-E_{n}^{\kappa })t}$.

Now if we take γ→∞, then the off-diagonal elements $H_{mn}^{\epsilon \kappa }$ vanish, but the diagonal elements are given by: $H_{n}^{\pm \pm }=\frac{1}{2}P_{n}P_{n}$. Therefore, the qubit and the cavity field end up in a separable (non-entangled) state: $\hat{R}\left(\gamma \to \infty \right)=\frac{1}{2}(|1\rangle \langle 1|+|0\rangle \langle 0|)⊗|\zeta_{S}\rangle \langle \zeta_{S}|$. The qubit is in a statistically mixed state while the cavity returns to the state $|\zeta_{S}\rangle$.

In the following section we use the density matrix elements derived here to study the dynamical properties of entropies and entanglement taking into account the cavity phase damping.

## 3. Qubit-Cavity Entanglement Dynamics

Here, we employ the negativity to quantify the qubit-cavity entanglement. For an arbitrary dimension (m⊗n)-quantum state (m<<n)$\hat{R}\left(t\right)$, the negativity is defined by [50,51],

(14) $$N\left(t\right)=\frac{1}{m-1}[∥{\hat{R}}^{PT}{∥}_{1}-1].$$

where $∥{\hat{R}}^{PT}{∥}_{1}$ represents the trace norm of the partial transpose matrix ${\hat{R}}^{PT}$ of the state $\hat{R}$ with respect to the qubit subsystem. The negativity can be generalized for qubits (n>2) instead of qubits [50] to analyze quantitatively the entanglement between their sub-systems. Consequently, the negativity is used to investigate the entanglement in (2⊗n)-[52] and (3⊗n)-quantum systems.

Based on the cavity basis {|n〉}(n=0,1,2,...), we define the cavity matrices ${\hat{M}}_{ij}=\langle i|\hat{R}\left(t\right)|j\rangle \;\left(i,j=1,0\right)$, and represent the qubit-cavity density matrix of Equation (13) as:

(15) $$\hat{R}\left(t\right)=\left(\begin{array}{cc} {\hat{M}}_{11} & {\hat{M}}_{10}\\ {\hat{M}}_{01} & {\hat{M}}_{00} \end{array}\right).$$

Therefore, the partial transpose matrix ${\hat{R}}^{PT}\left(t\right)$ of the qubit-cavity state $\hat{R}\left(t\right)$ in the basis {|1〉,|0〉} is defined by

(16) $${\hat{R}}^{PT}\left(t\right)=\left(\begin{array}{cc} {\hat{M}}_{11} & {\hat{M}}_{01}\\ {\hat{M}}_{10} & {\hat{M}}_{00} \end{array}\right).$$

By using the negative eigenvalues $\mu_{k}$ of the partial transpose matrix, the qubit-cavity negativity is given by:

(17) $$N\left(t\right)=-\sum_{k}\mu_{k}.$$

If N(t)=0.5, then the qubit-cavity system is in a maximally entangled state while N(t)=0 means that the system is in a disentangled state. Otherwise, for 0<N(t)<0.5, the qubit-cavity state has partial entanglement.

### 3.1. Case of $\hat{Y}\left({\hat{\psi }}^{†}\hat{\psi }\right)=\hat{I}$

**Figure 1:**
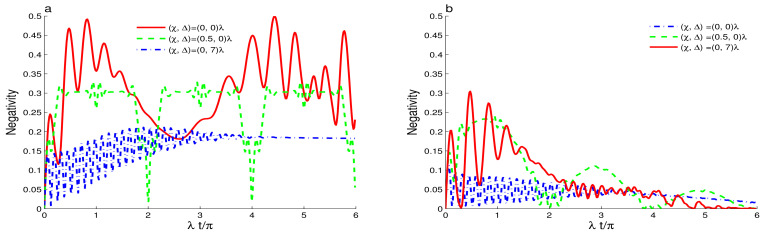
Dynamics of the entanglement for r=0.5, $\hat{Y}\left({\hat{\psi }}^{†}\hat{\psi }\right)=\hat{I}$, and the mean photon number ${\left|\zeta \right|}^{2}=16$ when the cavity field is initially in squeezed coherent state with different values of the Kerr-like nonlinearity and qubit-cavity detunings. For γ=0 in (**a**) and γ=0.1λ in (**b**).

Figure 1a shows of the generated amount of the qubit-cavity entanglement when the intensity-dependent coupling is constant $\hat{Y}\left(n\right)=1$ and the cavity field is initially in squeezed coherent state with different values of the Kerr-like nonlinearity. For the vanishing Kerr-like nonlinearity, the qubit-cavity detuning and the phase-damping, the qubit-cavity interaction generates a partial and maximal qubit-cavity entanglement with stochastic oscillations. Dashed curve of Figure 1a proves that the Kerr-like nonlinearity reduces the amplitudes of the negativity oscillations. The qubit-cavity entanglement is more stable and oscillate with 2π-period. For the off-resonant case Δ=7λ, see dashed-dotted curve of Figure 1a, we find that the enhancement of the qubit-cavity detuning decreases the upper values of the negativity. During the time interval λt∈[0,4π], the negativity rapidly oscillates with high frequencies. For λt>4π, dashed-dotted curve of Figure 1b shows that the negativity is time-independent, i.e., the qubit-cavity system reaches a partially entangled state. This generated time-independent entangled state can be used to implement quantum computation [9].

**Figure 2:**
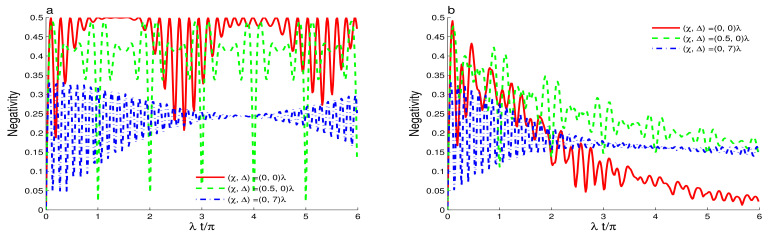
Qubit-cavity entanglement dynamics for r=0.5, $\hat{Y}\left({\hat{\psi }}^{†}\hat{\psi }\right)=\hat{I}$, and ${\left|\zeta \right|}^{2}=16$ when the cavity field is initially in the even squeezed coherent state with different values of the Kerr-like nonlinearity and the detuning. For γ=0 in (**a**) and γ=0.1λ in (**b**).

Figure 1b shows the effect of the phase cavity decoherence γ=0.1λ on the qubit-cavity entanglement dynamics. The rising of the phase coupling to the environment reduces the amplitudes of the negativity oscillations. After a particular time, the qubit-cavity entanglement vanishes. This means that the qubit and the cavity field are disentangled due to the increase of the phase decoherence. After a particular time, the dashed and dashed-dotted curves show that the robustness of the generated qubit-cavity entanglement can be enhanced in the presence of the Kerr-like nonlinearity and the qubit-cavity detuning. The vanishing of the qubit-cavity entanglement, due to the phase decoherence, can be delayed. For the Kerr-like nonlinearity χ>0, the green dashed plot shows that the phenomena of the sudden death and birth in the entanglement dynamics occur around λt/π=4 [53,54,55,56]. Figure 2 shows the effect of the initial cavity non-classicality by considering that the cavity is initially in the even superposition squeezed coherent state s=1 with the same the parameter values of Figure 1. We note that the high squeezed coherent non-classicality enhances the strength and the stability of the generated entanglement compared to the one generated with the coherent state. For the resonant case, the negativity has regular oscillatory behavior. Solid curve of Figure 2a shows that the qubit-cavity state can be stabilized in a maximally entangled state in particular time intervals in which N(t)=0.5. Dashed curve of Figure 2a confirms the effect of the Kerr-like nonlinearity that is observed in the previous cases. The amplitudes of the negativity decrease. The qubit-cavity entanglement oscillates with a π-period, and its amount is less than the one of the case s=0. The initial even coherent states (s=1) induce stronger entanglement. Figure 2b illustrates the combined effects of the phase cavity damping, the Kerr-like, and the detuning as well as the high initial field non-classicality. The negativity is plotted with the same parameter values of Figure 2a but for γ=0.1λ. We find that the high squeezed coherent non-classicality reduces the effect of the phase-damping, and inhibits the vanishing qubit-cavity entanglement. It is more robust against the phase-damping with the increase of the Kerr-like nonlinearity and the detuning.

### 3.2. Case of $\hat{Y}\left({\hat{\psi }}^{†}\hat{\psi }\right)=\sqrt{{\hat{\psi }}^{†}\hat{\psi }}$

**Figure 3:**
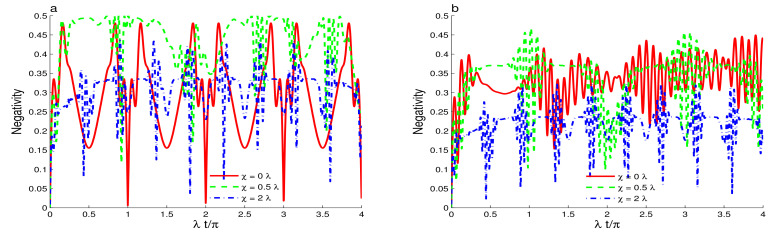
Dynamics of the entanglement for r=0.5, ${\left|\zeta \right|}^{2}=16$, $\hat{Y}\left({\hat{\psi }}^{†}\hat{\psi }\right)=\sqrt{{\hat{\psi }}^{†}\hat{\psi }}$ with different values of the Kerr-like nonlinearity in the absence of the dissipative phase-damping. We use Δ=0 in (**a**) and Δ=7λ in (**b**).

**Figure 4:**
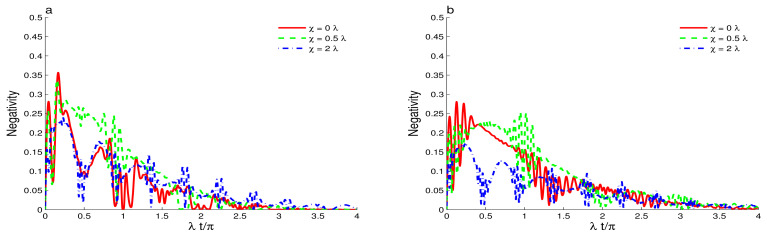
Dynamics of the entanglement for r=0.5, ${\left|\zeta \right|}^{2}=16$, $\hat{Y}\left({\hat{\psi }}^{†}\hat{\psi }\right)=\sqrt{{\hat{\psi }}^{†}\hat{\psi }}$ with different values of the Kerr-like nonlinearity for the phase-damping coefficient, γ=0.2λ. We use Δ=0 in (**a**) and Δ=7λ in (**b**).

**Figure 5:**
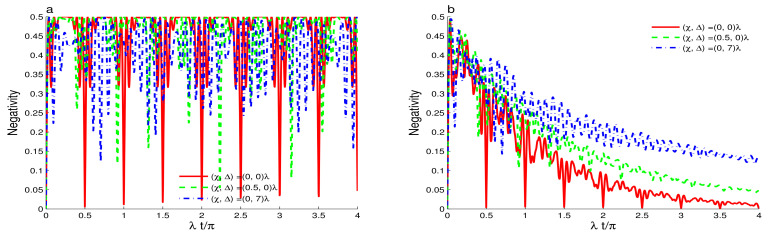
Negativity dynamics for r=0.5, ${\left|\zeta \right|}^{2}=16$, $\hat{Y}\left({\hat{\psi }}^{†}\hat{\psi }\right)=\sqrt{{\hat{\psi }}^{†}\hat{\psi }}$ and the squeezed even coherent state with different values of the Kerr-like nonlinearity and the qubit-cavity detuning. We use γ=0 in (**a**) and γ=0.2λ in (**b**).

Figure 3, Figure 4 and Figure 5 shows of the generated amount of the qubit-cavity entanglement when the intensity-dependent coupling is described by the function $\hat{Y}\left({\hat{\psi }}^{†}\hat{\psi }\right)=\sqrt{{\hat{\psi }}^{†}\hat{\psi }}$ and the cavity field is initially in different squeezed coherent states with different values of the Kerr-like nonlinearity and the detuning as well as the phase cavity decoherence. By comparing Figure 1a and Figure 3a, where the effects of the Kerr-like medium, the detuning and the damping are absent, we find that the qubit-cavity interaction through the intensity-dependent coupling generates π-periodic entanglement. Dashed curves show that the small value of the Kerr-like nonlinearity enhances the entanglement with irregular oscillatory behavior. This entanglement can be stabilized during a short time. For a large value of the Kerr-like medium, see dashed-dotted curve, the amplitudes of the negativity is reduced with additional irregular fluctuations.

Figure 3b shows the effect of the higher detuning Δ=7λ in the presence of the intensity-dependent coupling. We find that the detuning reduces the amplitudes of the negativity oscillations with rapid stochastic oscillations.

**Figure 6:**
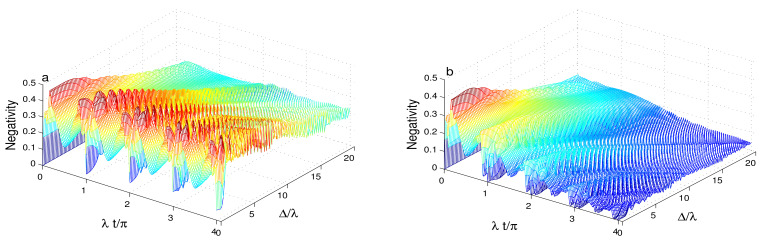
Entanglement dynamics for r=0.5, χ=0, ${\left|\zeta \right|}^{2}=16$, $\hat{Y}\left({\hat{\psi }}^{†}\hat{\psi }\right)=\sqrt{{\hat{\psi }}^{†}\hat{\psi }}$ when the squeezed coherent state s=0 with different qubit-cavity detuning values Δ∈[0,20]λ. We use γ=0 in (**a**) and γ=0.1λ in (**b**).

In Figure 4, we note that the phase cavity damping reduces the entanglement in the presence of the intensity-dependent coupling. In this case, the intensity-dependent coupling enhances the sensitivity of the generated entanglement to the effect of the phase-damping. The qubit-cavity entanglement is deteriorated rapidly compared to the case where $\hat{Y}\left({\hat{\psi }}^{†}\hat{\psi }\right)=\hat{I}$. In Figure 4a, the dashed and solid curves demonstrate that the entanglement phenomena of sudden death and birth can occur. The phenomena disappear due to the increase of the Kerr-like nonlinearity and the qubit-cavity detuning. Figure 4b confirms that after a particular time, the Kerr-like nonlinearity and the qubit-cavity detuning enhance the robustness of the generated entanglement against the phase-damping. Figure 5 illustrates the dependence of the qubit-cavity entanglement (in the presence of the Kerr-like medium, the detuning and the damping) for an even coherent state as initial state. Please note that the high cavity non-classicality leads to notable changes in the qubit-cavity entanglement dynamics as: (1) The stability intervals of the generated maximally entangled qubit-cavity state is strengthened, see solid and dashed curves of Figure 5a. (2) By comparing the effects of the initial states for different non-classicality (s=1 and s=1), we observe that for s=1, the periodicity of the entanglement oscillations are reduced to 0.5π. (3) The different effects of the Kerr-like nonlinearity and the detuning against the phase-damping are enhanced. The high initial cavity non-classicality enhances the robustness of the qubit-cavity entanglement against the phase-damping effect. Figure 6 illustrates how the entanglement depends on the qubit-cavity detuning Δ∈[0,20]λ. From Figure 6a, we note that for Δ∈[0,5]λ, the qubit-cavity state has a higher degree of entanglement. After that, the increase of the detuning reduces the negativity entanglement. The entanglement stability is enhanced by increasing the qubit-cavity detuning. When the phase-damping is raised, the negativity is reduced. The qubit-cavity state reaches a non-entangled one, see Figure 6b. The reduction of the entanglement is accelerated as the qubit-cavity detuning is enhanced.

**Figure 7:**
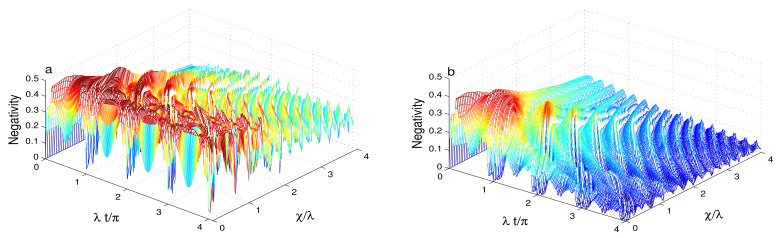
Entanglement dynamics for r=0.5, Δ=0, ${\left|\zeta \right|}^{2}=16$, $\hat{Y}\left({\hat{\psi }}^{†}\hat{\psi }\right)=\sqrt{{\hat{\psi }}^{†}\hat{\psi }}$ when the squeezed coherent state s=0 with different Kerr-like nonlinearity values χ∈[0,2]λ. We use γ=0 in (**a**) and γ=0.1λ in (**b**).

Figure 7 shows the dependence of the entanglement on the Kerr-like nonlinearity χ∈[0,4]λ. Please note that for small Kerr-like nonlinearity values χ∈[0,2]λ, the qubit-cavity negativity shows a high degree of entanglement, see Figure 7a. Figure 7b demonstrates that the reduction of the entanglement induced by the phase cavity damping effect can be realized faster by increasing the Kerr-like nonlinearity.

**Figure 8:**
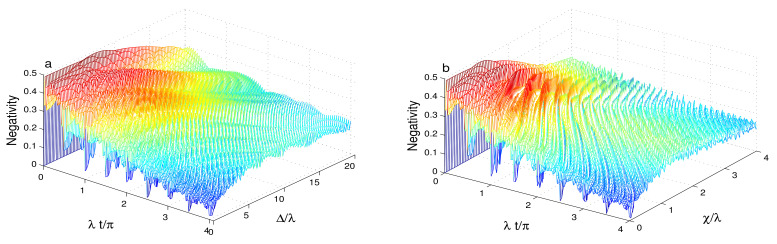
Qubit-cavity entanglement dynamics of the same parameter values of Figure 6b and Figure 7b are in (**a**) and (**b**), respectively, but when the cavity field is initially in the even superposition squeezed coherent state.

Figure 8 shows the effect of the initial cavity non-classicality of the even squeezed coherent state s=1 on the reduced entanglement entanglement due to the phase cavity damping.

By comparing Figure 6b and Figure 7b with Figure 8, we find the high-squeezed even-coherent non-classicality enhances the degree and the stability of the generated entanglement. According to Figure 8, the reduced entanglement and its stability are more sensitive to the Kerr-like nonlinearity than to the qubit-cavity detuning.

## 4. Conclusions

In this work, we analytically solved the master equation of the phase cavity damping effect to explore the dynamics of a qubit interacting off-resonantly with a nonlinear Kerr-like cavity with intensity-dependent coupling. Based on the negativity, the dynamics of the entanglement between the qubit and the cavity is investigated when the cavity field is initially in a superposition of coherent squeezed coherent states. For different cases of the intensity-dependent coupling, the generated entanglement is explored under the effects of the Kerr-like nonlinearity, qubit-cavity detuning, the phase-damping as well as for two different initial coherent states. It is found that the stability and the degree of the generated entanglement can be controlled by the Kerr-like nonlinearity, the qubit-cavity detuning, and the initial cavity states.

The small values of the Kerr-like nonlinearity and the qubit-cavity detuning enhance the generated qubit-cavity entanglement due to their interactions. However, for large values of the Kerr-like medium and detuning, the entanglement stability is accelerated. By comparing the degree, robustness, and stability of the entanglement generated with the coherent state, the squeezed even coherent state improves the entanglement’s resistance to phase-damping. We have also observed that the intensity-dependent coupling increases the sensitivity of the generated entanglement to the phase-damping.

## Data Availability

Not applicable.
